# Lower hamstring extensibility in men compared to women is explained by differences in stretch tolerance

**DOI:** 10.1186/1471-2474-15-223

**Published:** 2014-07-07

**Authors:** Paul WM Marshall, Jason C Siegler

**Affiliations:** 1 School of Science and Health, University of Western Sydney, Locked Bag 1797, Penrith South, NSW 2751, Australia

**Keywords:** Hamstring extensibility, Passive stiffness, Stretch tolerance

## Abstract

**Background:**

This study examined whether passive hamstring tissue stiffness and/or stretch tolerance explain the relationship between sex and hamstring extensibility.

**Methods:**

Ninety healthy participants, 45 men and 45 women (mean ± SD; age 24.6 ± 5.9 years, height 1.72 ± 0.09 m, weight 74.6 ± 14.1 kg) volunteered for this study. The instrumented straight leg raise was used to determine hamstring extensibility and allow measurement of stiffness and stretch tolerance (visual analog pain score, VAS).

**Results:**

Hamstring extensibility was 9.9° greater in women compared to men (p = 0.003). VAS scores were 16 mm lower in women (p = 0.001). Maximal stiffness (maximal applied torque) was not different between men and women (p = 0.42). Passive stiffness (slope from 20-50° hip flexion) was 0.09 Nm.°^-1^ lower in women (p = 0.025). For women, linear and stepwise regression showed that no predictor variables were associated with hamstring extensibility (adjusted r^2^ = -0.03, p = 0.61). For men, 44% of the variance in hamstring extensibility was explained by VAS and maximal applied torque (adjusted r^2^ = 0.44, p < 0.001), with 41% of the model accounted for by the relationship between higher VAS scores and lower extensibility (standardized β coefficient = -0.64, p < 0.001).

**Conclusions:**

The results of this study suggest that stretch tolerance and not passive stiffness explains hamstring extensibility, but this relationship is only manifest in men.

## Background

Extensibility is defined as the ability of muscle tissue to lengthen or stretch beyond resting length. Lower hamstring extensibility is a functional characteristic of significant interest for the prevention and rehabilitation of locomotion related strain injuries [1,2], as well as the treatment of patients with chronic low back pain [3-5]. Passive stretching is a typical component of injury prevention and rehabilitation programs to address hamstring extensibility [6]. However, there is dispute as to the efficacy of passive stretching for improving hamstring extensibility. The equivocal evidence may, in part, be attributed to the use of different volumes of stretching prescribed between studies [7-9], small sample sizes, confusion about the mechanism of action for explaining changes in hamstring extensibility, and the confounding effect of small mixed sex groups [10-12]. Two factors typically measured for explaining changes in hamstring extensibility are passive stiffness and stretch tolerance. There is debate regarding the relative contribution of passive stiffness and stretch tolerance to hamstring extensibility. Moreover there is limited information examining similarities or differences between-sex in passive stiffness and stretch tolerance. The limited evidence examining between-sex differences contributes to the confusing evidence from training studies that use small mixed sex groups to examine changes in hamstring extensibility.

Muscle stiffness is the ratio of the change in torque to the change in muscle length, which may be examined during active or passive contractions [13]. Often measured during an instrumented passive straight leg raise (iSLR) test to allow observation of torque applied during stretch [11,14,15], some research that has concluded passive stiffness does not explain extensibility, either acutely or following training [8,10,11,16]. However, these studies typically define passive stiffness from the maximal torque measured at the end of the hamstrings range of motion [8,10,11,16]. Maximal applied torque is probably not a valid estimation of passive muscle stiffness as the measurement does not take into account the ratio of the change in torque to change in muscle length during stretch. In contrast increased passive hamstring stiffness, quantified as the slope of the torque-angular position curve during passive hip flexion, was observed to significantly predict decreased extensibility in both healthy individuals and patients with chronic LBP [5]. Moreover, reductions in the slope of the torque-angular position curve were associated with improved hamstring extensibility following 4-weeks of passive stretching in healthy young men and women [12]. The reliance on measurement of maximal torque rather than examining changes in torque during the range of motion has contributed to the conclusion that hamstring extensibility is probably best explained by stretch tolerance.

Stretch tolerance is described as the willingness to tolerate the discomfort associated with stretch [11,17]. Typically, this has been self-reported as the angle during the range of motion where discomfort is reported [8,17]. One study suggested stretch tolerance does not explain extensibility, but no direct measure of stretch tolerance was performed during testing [5]. Other evidence that suggests stretch tolerance may contribute to hamstring extensibility has examined maximal perceived pain during an extensibility test before and after training interventions [11,12]. While increased hamstring extensibility was reported following passive stretch training programs [11,12], maximal pain reported at the new end range of extensibility was not changed. This was interpreted in one study to mean that participants willingness to tolerate pain during stretch had improved [11], and was not discussed in the other [12]. Stretch tolerance does likely predict extensibility, but the contribution to extensibility as compared to changes in passive stiffness is unknown. Moreover, it is unclear whether there are between-sex differences in the contribution of stretch tolerance and passive stiffness to total hamstring extensibility.

One study using the iSLR reported no between-sex difference for extensibility, but lower passive stiffness for women [5]. This finding [5] is confounded by a small, mixed sample of healthy controls and LBP patients, and absence of a direct stretch tolerance measure. Other evidence using a prone knee flexion/extension perturbation model reported lower active and passive hamstring stiffness in women [18,19]. Thus it is reasonable to believe that passive stiffness will be lower in women, although it is not clear whether this will explain differences in extensibility during the iSLR. Moreover, it is unclear whether or not stretch tolerance will be lower in women concomitant to lower passive stiffness. Differences between men and women for pain sensitivity and tolerance have been reported [20]. Typically, these findings suggest that women have greater pain sensitivity and lower tolerance than men, which is often attributed to different hormonal profiles [20-22]. Whether women will self-report greater pain during stretch, when it is expected that passive stiffness will be lower and extensibility higher than men is unclear.

There is a need to understand whether the relationship between sex and hamstring extensibility is explained by hamstring passive tissue stiffness and stretch tolerance. Providing information about the factors that contribute to hamstring extensibility within each sex will help the design of targeted interventions. The purposes of this study were: 1) to compare measures of hamstring extensibility, passive stiffness, and stretch tolerance between men and women, 2) to examine the relationship between passive stiffness and stretch tolerance with hamstring extensibility within each sex.

## Methods

### Participants

Ninety participants, 45 men and 45 women, from a university population volunteered for this study (mean ± SD, men, age 23.4 ± 4.5 years, height 1.79 ± 0.05 m, mass 84.2 ± 10.3 kg; women, age 25.7 ± 6.9 years, height 1.65 ± 0.06 m, weight 64.9 ± 10.2 kg). Men were taller and heavier (p < 0.05). Participants were required to be free from any known metabolic and neuromuscular disease, or have any lifetime history of hamstring strain injury. Lifetime hamstring strain injury was defined as any hamstring injury that required absence from work, sport, training, or pain that required medication or treatment by a health care professional. No participant could recall an acute episode or period of back or hip pain where they had to seek medical treatment or miss a day of work or exercise. Current physical activity participation was collected from all participants via self-reporting (n = 42 participants reported no regular physical activity apart from normal daily activities). For physically active participants, most reported performing various modes of exercise which did not make it possible to classify their training into one category for purposes of statistical analysis (e.g. yoga, stretching, resistance exercise). The two delineating factors between sexes for physical activity levels were that more men reported performing regular resistance exercise (n = 10 reported between two to six resistance based sessions per week) compared to women (n = 3), while more women reported regular (n = 11) participation in group based exercise sessions (e.g. step aerobics, spin classes) compared to men (n = 0). Similar numbers of men (n = 12) and women (n = 15) reported performing at least one weekly stretching session (e.g. home based stretching, stretching prior to sport training session, yoga or pilates class). Written informed consent was received from all participants. Ethical approval was received for this study from the University Human Research Ethics Committee.

### Instrumented straight leg raise (iSLR)

The KinCom isokinetic dynamometer (Chattanooga, KinCom 125 Version 5.32) was used for iSLR testing. Participants were placed in a supine position on the examination table. They were fixed to the table by the use of straps across the trunk, pelvis, and opposite thigh. Adjustable support was provided to prevent the lumbar spine flattening onto the test surface during testing. The leg was fully extended in the apparatus and attached to the lever arm on the distal aspect of the thigh and mid-shank, and the ankle was fixed at 90° of dorsiflexion. The center of rotation of the dynamometer lever arm was aligned with the hip joint center of rotation through the greater trochanter of the test limb. Following familiarization with the experimental set-up and preliminary movement through a partial range of motion (passive flexion of the hip performed by the investigator), the stop angle to be used during testing was established by passively flexing the participant’s hip until they could no longer tolerate the stretch. This was performed between 3 to 5 times with 30 s rest between movements until a consistent maximum hip flexion angle (within 1° hip flexion as observed from digital real-time output of the dynamometer software) was established. The maximum angle of these trials was set as the terminal limit of the dynamometer for the recorded trial. This procedure was used to ensure that a true maximum hip flexion angle was set, reducing the influence of participant choice on termination of range of motion. The hip was then passively flexed through the established range of motion by the dynamometer at a movement velocity of 5°.sec^-1^, which is similar to previous studies [5,15,17].

### Hamstring extensibility

Angular position of the lever arm and torque were continuously collected at 100Hz. Maximum hip flexion angle (leg°_max_) in the sagittal plane was measured from output of the angular position of the lever arm relative to the initial starting position (leg fully extended in line with the trunk was calibrated to starting angle of 0°). Therefore leg°_max_ was the dependent variable we measured and defined as representing hamstring extensibility. Inter-trial reliability within this study for leg°_max_ during the iSLR was high (ICC r = 0.98). Intra-tester reliability for the iSLR has been reported (ICC r = 0.94, [23]), in addition to between-session reliability (ICC r = 0.95, [12]).

### Hamstring stiffness

**Figure 1:**
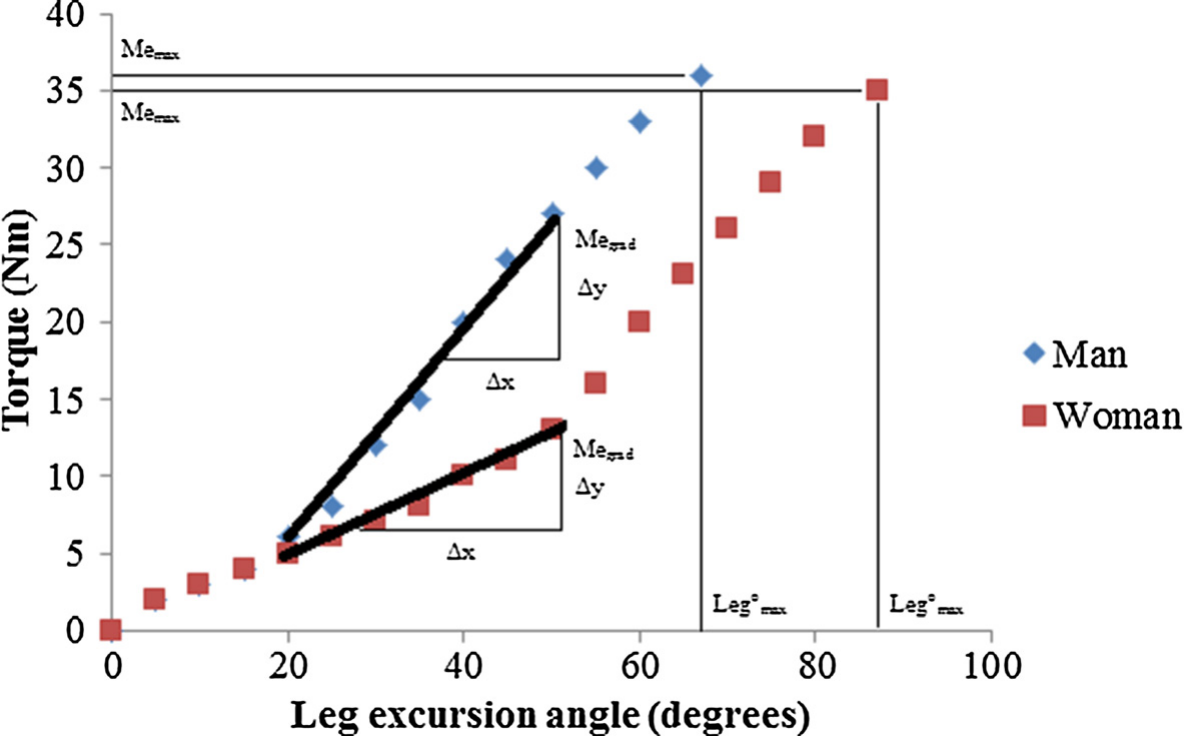
**Representative results from a man and woman tested in this study for the torque measured during the instrumented straight leg raise test (iSLR).** Passive tissue stiffness was measured as the slope (∆y/∆x) through the common range of motion (Me_grad_, 20-50°), and peak torque applied during the iSLR (Me_max_). Maximum leg excursion angle (leg°_max_) was used to represent hamstring extensibility in this study (male, 67°; female, 87°). Observe the lower Me_grad_, and greater leg°_max_ for the female participant, but similar Me_max_. Note that VAS pain scores were 66 and 4 mm respectively for the male and female participant results presented here.

The active torque required to lift the leg was continuously recorded (Figure 1) and adjusted for limb weight, leg length, and the angular position of the lever arm. Stiffness was defined as the torque calculated at the different joint angles (Me). The maximal value from the torque-time curve was recorded and defined as the maximal stiffness (Me_max_). The relative stiffness (Me_grad_) through the common range of motion (20 to 50° based on hip flexion angle) was calculated from the slope of a linear trend line fitted to torque values recorded from 20 to 50° hip flexion. For processing Me_grad,_ recorded torque values in 5° increments (20°, 25°…..45°, 50°) were calculated from the average 1-s torque recorded at each point in the range of motion (e.g. average torque recorded from 19.5 to 20.5° was calculated for stiffness at 20° hip flexion). Subsequently, the slope (Δtorque/Δangular position; Nm.°^-1^) and coefficient of determination (r^2^) to examine the goodness of fit were calculated from a linear line fit to the torque-angular position relationship for each participant. The common range of motion is achievable in healthy and clinical populations [5,24]. The average coefficient of determination for the linear line for participants was r^2^ = 0.94 ± 0.01 (not different between men and women). Analysis through the common range of motion allows comparison of passive tissue stiffness across a standard absolute range of motion between participants, independent of total extensibility or maximal applied torque, and is a measure that allows direct comparison across population cohorts with lower absolute extensibility (e.g. low back pain patients, athletes pre-season with low extensibility).

### Stretch tolerance

The visual analog pain scale (VAS) was used to represent participant stretch tolerance. The VAS is a 100 mm horizontal line with “no pain” and “worst pain” anchored to the respective left (0 mm) and right (100 mm) ends. Participants were asked to draw a vertical line through the horizontal line at the point which best represented their maximal pain intensity experienced during the iSLR. The VAS score (mm) was calculated by measuring the distance from “no pain” to their mark. The VAS score was collected immediately (<5 s) following iSLR testing. Between-trial reliability for VAS scores reported during the manual SLR tests to establish maximum range of motion (performed prior to the recorded iSLR) had an ICC r-value > 0.95. Test-retest reliability of the VAS to measure self-rated pain has been demonstrated to be high with ICC scores between 0.70 and 0.83 [25]. The VAS has been estimated to be more sensitive to change than a verbal rating scale, and similar to an 11-point numeric rating scale [26].

### Statistical analysis

Data were analyzed using SPSS v20 (Armonk NY, USA: IBM Corp). All data were normally distributed, as assessed from inspection of the skewness and kurtosis of the data and Kolmogorov-Smirnov normality tests. Dependent variables were compared between men and women using independent samples t-tests. Mean differences and 95% confidence intervals were calculated for between-sex differences. Pearson’s correlation coefficients were calculated for within-sex relationships between the predictor variables Me_max,_ Me_grad,_ and VAS. Multiple linear and step-wise regression analyses were used to examine the relationship between Me_max,_ Me_grad,_ and VAS scores with leg°_max._ Regression analyses were performed for the entire sample, and separately for each sex. The significance level for this study was p < 0.05.

## Results

Men and women results for iSLR variables are presented in Table 1. Leg°_max_ was 9.9° greater in women compared to men (95% CI for mean difference, 3.5 to 16.3°, p = 0.003). VAS scores were 16 mm lower in women (95% CI for mean difference, -7 to -26 mm, p = 0.001). Me_grad_ was 0.09 Nm.°^-1^ lower in women (95% CI for mean difference, -0.01 to -0.16 Nm.°^-1^, p = 0.025). No between-sex difference was observed for Me_max_.

**Table 1:** **Between sex results (mean ± SD, lower and upper limits for 95% confidence interval, CI) for iSLR measures of hamstring extensibility (leg°**_**max**_**), passive stiffness (Me**_**max**_**, Me**_**grad**_**), and stretch tolerance (hamstring VAS;mm)**

**Variable**	**Men (n = 45)**		**Women (n = 45)**		**p-value**
	**Mean ± SD**	**95% CI**	**Mean ± SD**	**95% CI**	
**leg°**_**max **_**(°)**	79.4 ± 17.8	73.5 to 85.4	89.4 ± 12.4	85.2 to 93.5	0.003
**Me**_**max **_**(Nm)**	43.6 ± 23.6	35.7 to 51.5	40.3 ± 14.6	35.4 to 45.1	0.42
**Me**_**grad **_**(Nm.°**^**-1**^**)**	0.47 ± 0.15	0.42 to 0.53	0.38 ± 0.21	0.32 to 0.46	0.025
**Hamstring VAS (mm)**	41 ± 26	32 to 49	24 ± 19	18 to 30	0.001

Between-sex differences were observed for leg°_max,_ Me_grad,_ and VAS (t-test p-values presented in table).

For women, correlation analysis identified no association between VAS and Me_max_ (r = 0.19, p = 0.21) or Me_grad_ (r = 0.12, p = 0.41), and a significant positive association between the respective passive stiffness measures (r = 0.47, p = 0.001). For men, there was a significant positive association between VAS and Me_max_ (r = 0.66, p < 0.001), but no association between VAS and Me_grad_ (r = 0.11, p = 0.47), or between the respective passive stiffness measures (r = 0.26, p = 0.10).

**Figure 2:**
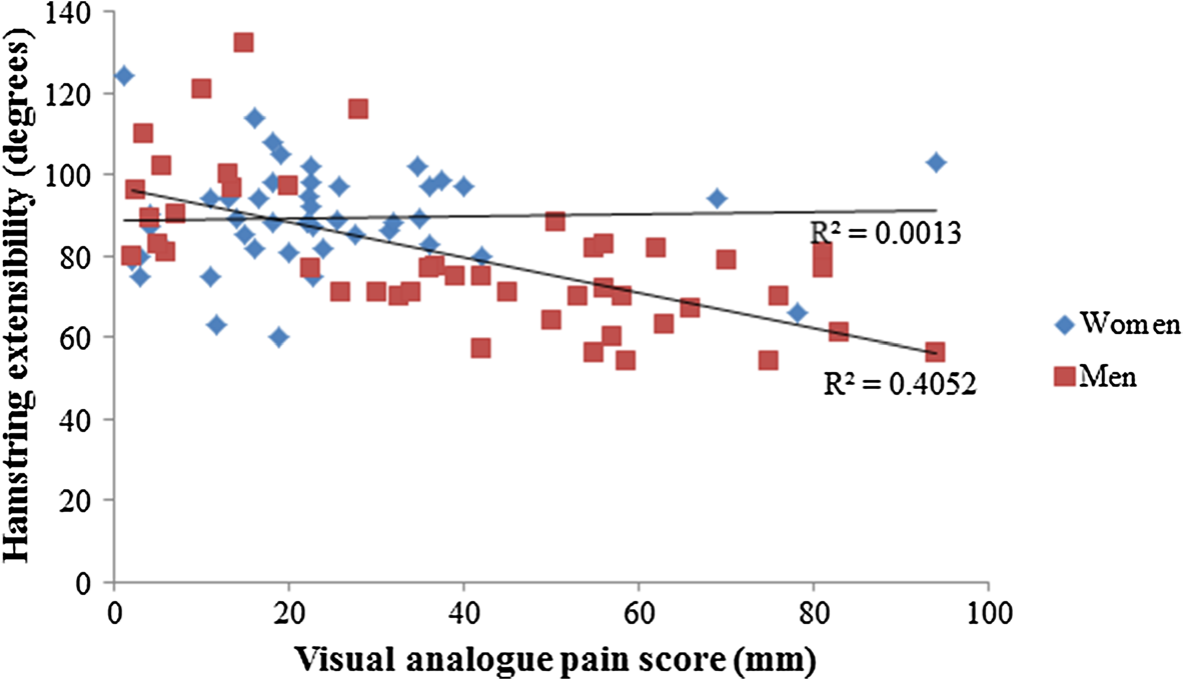
**Relationship between hamstring extensibility (degrees; °) and visual analog pain scores (VAS; mm) for men (n = 45) and women (45) in this study.** Trend lines are displayed for men and women, with a significant association between VAS and hamstring extensibility for men only (adjusted r2 = 0.41, standardized β coefficient = -0.64, p < 0.001).

For all participants, the predictor variables significantly explained 23% of the variance in Leg°_max_ (adjusted r^2^ = 0.23, p < 0.001). Stepwise regression identified VAS as the primary predictor of Leg°_max_ (adjusted r^2^ = 0.22, p < 0.001), with higher VAS scores associated with lower Leg°_max_ (standardized β coefficient = -0.48, p < 0.001). For women, linear and stepwise regression showed that no predictor variables were associated with Leg°_max_ (Figure 2; adjusted r^2^ = -0.03, p = 0.61). For men, 44% of the variance in Leg°_max_ was explained by the predictor variables (adjusted r^2^ = 0.44, p < 0.001). Stepwise regression revealed that VAS explained 41% of variance, with higher VAS scores associated with lower Leg°_max_ (Figure 2; standardized β coefficient = -0.64, p < 0.001), and Me_max_ contributed a further 3% to the model with higher scores associated higher Leg°_max_ (standardized β coefficient = -0.32, p = 0.041).

## Discussion

### Main findings

The main findings of this study were 1) women had lower passive stiffness through a common range of motion, better stretch tolerance (lower VAS scores), and greater hamstring extensibility compared to men, and 2) stretch tolerance, but not passive stiffness, was a significant predictor of total hamstring extensibility for men only.

### Between sex differences in extensibility

The results of this study support previous findings for between-sex differences in the mechanical properties of hamstring stiffness and extensibility [18,19]. Previous research has provided insight into mechanistic factors that may contribute to the between-sex differences reported here. One study reported that increased electromechanical delay (EMD) of the medial hamstrings during the iSLR test was associated with greater hamstring extensiblity in women, suggesting that the neuromuscular control of the hamstrings during stretch is altered in women [5]. Another study [18] reported that greater hamstring stiffness in men, as measured by an oscillating knee flexion/extension protocol, was positively associated with hamstring cross-sectional area (CSA). Measures of both stiffness and CSA were greater in male participants [18]. During the iSLR procedure in the current study, the applied torque to passively flex the hip was corrected for the limb weight, angular position, and lever arm of the participant. This calculation does not account for hamstring muscle CSA. Future research should examine whether passive stiffness, as measured by the iSLR method used in this study, may be explained by hamstring CSA and thus explain between-sex differences observed.

### The association between stretch tolerance and extensiblity

The results of this study provide evidence that stretch tolerance, defined as the intensity of pain elicited during stretch, is the main explanatory variable of hamstring extensibilty as compared to measures of passive stiffness. This relationship was only observed for men, and not women. Lower VAS scores in women was unexpected, as previous findings suggest that women have higher pain sensitivity [20-22]. The self-report of pain, as performed in this study, is a complex interaction between physiological inputs, particularly nociceptive input, and behavioural interpretation. Higher pain scores in men may be associated with between sex differences in afferent feedback, particularly of the group III afferents. Group III afferents, also known as Aδ fibers, are thinly myelinated fibers and have free nerve endings within the connective tissue of skeletal muscle [27]. Group III afferents primarily transmit information about mechanical stimuli in muscle [28], and in combination with group IV afferents are activated by nociceptive stimuli (e.g. bradykinin) and thus are the proposed source of pain in skeletal muscle. Research examining discharge properties of group III afferents in response to constant stretch is equivocal, but responses do increase as tension developed in a muscle increases [29]. It is not clear whether the pain scores reported in this study, particularly those reported by men, are mediated by increased group III afferent discharge rates. Moreover, there is no evidence examining group III afferent discharge properties during a stretching task where mechanical tension within the muscle is gradually increasing, or comparing group III discharge properties between sexes. Concomitant to nociception potentially explaining between sex differences in stretch tolerance is consideration for whether pain is causing a reflex response that impairs hamstring extensibility in men.

Johansson and Sojka proposed that muscle pain produces disturbances in proprioception, stiffness regulation, and motor control by altering stretch sensitivity and the discharge of spindle afferents via gamma fusimotor neurons [30]. Thus, it is reasonable to believe that increased pain during stretch may alter the neuromuscular control of the hamstrings, increase the eccentric contraction during stretch, and limit extensibility. Existing evidence would suggest that muscle activity does not contribute to termination of the straight leg raise. It was recently reported that surface EMG signals obtained from the hamstring muscles during the passive straight leg raise were not related to measures of stiffness or extensibility [5]. Indeed, healthy controls exhibited higher hamstring EMG signals as well as greater extensibility compared to patients with chronic low back pain, thus negating suggestions that increased muscle activity is detrimental during a passive straight leg raise. The physiological link between pain and increased spindle output has also been questioned. Recent evidence using experimentally induced muscle pain reported that muscle spindle output was not increased, suggesting that stimulation of group III and IV afferents fails to excite fusimotor neurons and increase muscle spindle discharge [31]. Therefore the causation for termination of the test and thus the relationship between pain and extensibility, evident only in men, is probably mediated by behavioural interpretation of the pain elicited during stretch.

### Application and limitations

Recommendations for training interventions to improve hamstring extensibility in each sex cannot be made in this study. While current evidence would suggest that higher volumes of stretching (e.g. multiple hamstring stretches performed up to 5 times per week) are most effective for increasing extensibility and thus should be prescribed to both sexes [7,12], it is not clear whether men also need additional intervention to improve their ability to tolerate stretch. The results of this study do suggest that findings and conclusions from previous literature exploring mechanisms of action for changing hamstring extensibility using small mixed sex samples are strongly confounded because of the differences between each sex for the relationship between stretch tolerance and extensibility.

There are several limitations to this study. First, while we recorded current physical activity levels, we did not record historical physical activity levels. Since the between-sex difference in extensibility was likely explained by pain and the behavioural response to pain, it is a noteworthy limitation that historical activity was not recorded. It is reasonable to believe that historical exposure to stretching, and types of activity where hamstring range of motion is trained (e.g. dance), could influence the familiarity with the type of stretch used in this study and thus the between-sex difference. Other muscles in the posterior kinetic chain (e.g. gluteals) likely contribute to the extensibility and stiffness measures in this study. Therefore, while the primary muscle stretch is applied to, these results are not specific to only the hamstrings. While not measured, we do not believe that hamstring extensibility was significantly affected by changes in the displacement or strain of the lumbosacral nerve roots during iSLR testing. By stabilizing the lumbar spine and using a fixed ankle position we have controlled these potentially confounding variables that have been observed to increase spinal nerve root tension [32]. Finally, the two measures of passive stiffness were based on the slope through the common range of motion (20 to 50° hip flexion) and the maximal applied torque. Other researchers have applied a 4^th^ order polynomial to hamstring torque data during stretch, and have calculated the average first differential from a number of common points during the range of motion as the measure of stiffness (e.g. every 5° during the final 15° during the test [33]). Polynomial modelling may adequately fit the entire torque-angular position curve, but absolute application of a 4^th^ order polynomial for all participants assumes this is the best fit for the data. The passive stiffness measure in this study is based on the assumption of linearity between torque and angular position (r^2^ = 0.94 ± 0.01) in a range of motion (20 to 50°) that is achievable for healthy and chronic back pain populations. In future, methods should be applied that are individualized to each participant for curve-fit modelling and quantifying stiffness.

## Conclusions

Greater hamstring extensibility in women was not associated with the lower passive stiffness and stretch tolerance scores. In contrast, lower extensibility in men was associated with higher pain scores. The results of this study suggest that stretch tolerance does explain hamstring extensibility, but this relationship is only manifest in men.

## Competing interests

The authors declare that they have no competing interests.

## Author contributions

PM designed the study and coordinated data collection and analysis. PM and JS were both involved in the data analysis, preparation and editing of the final manuscript. Both authors read and approved the final manuscript.

## Pre-publication history

The pre-publication history for this paper can be accessed here:

http://www.biomedcentral.com/1471-2474/15/223/prepub
